# An unusual ectopic thymoma clonal evolution analysis: A case report

**DOI:** 10.1515/biol-2022-0600

**Published:** 2023-05-18

**Authors:** Sijia Zhang, Lu Wu, Zhenyu Li, Qianwen Li, Yan Zong, Kuikui Zhu, Leichong Chen, Haifeng Qin, Rui Meng

**Affiliations:** Cancer Center, Union Hospital, Tongji Medical College, Huazhong University of Science and Technology, No. 156 Wujiadun, Jianghan District, Wuhan, Hubei Province, 430022, China; Department of Pulmonary Neoplasm Internal Medicine, Fifth Medical Center of Chinese PLA General Hospital, Beijing, 100071, China

**Keywords:** ectopic pulmonary thymoma, immunohistochemical, heterogeneity, clonal evolution, case report

## Abstract

Thymomas and thymic carcinomas are rare and primary tumors of the mediastinum which is derived from the thymic epithelium. Thymomas are the most common primary anterior mediastinal tumor, while ectopic thymomas are rarer. Mutational profiles of ectopic thymomas may help expand our understanding of the occurrence and treatment options of these tumors. In this report, we sought to elucidate the mutational profiles of two ectopic thymoma nodules to gain deeper understanding of the molecular genetic information of this rare tumor and to provide guidance treatment options. We presented a case of 62-year-old male patient with a postoperative pathological diagnosis of type A mediastinal thymoma and ectopic pulmonary thymoma. After mediastinal lesion resection and thoracoscopic lung wedge resection, the mediastinal thymoma was completely removed, and the patient recovered from the surgery and no recurrence was found by examination until now. Whole exome sequencing was performed on both mediastinal thymoma and ectopic pulmonary thymoma tissue samples of the patient and clonal evolution analysis were further conducted to analyze the genetic characteristics. We identified eight gene mutations that were co-mutated in both lesions. Consistent with a previous exome sequencing analysis of thymic epithelial tumor, *HRAS* was also observed in both mediastinal lesion and lung lesion tissues. We also evaluated the intratumor heterogeneity of non-silent mutations. The results showed that the mediastinal lesion tissue has higher degree of heterogeneity and the lung lesion tissue has relatively low amount of variant heterogeneity in the detected variants. Through pathology and genomics sequencing detection, we initially revealed the genetic differences between mediastinal thymoma and ectopic thymoma, and clonal evolution analysis showed that these two lesions originated from multi-ancestral regions.

## Graphical abstract

**Figure:**
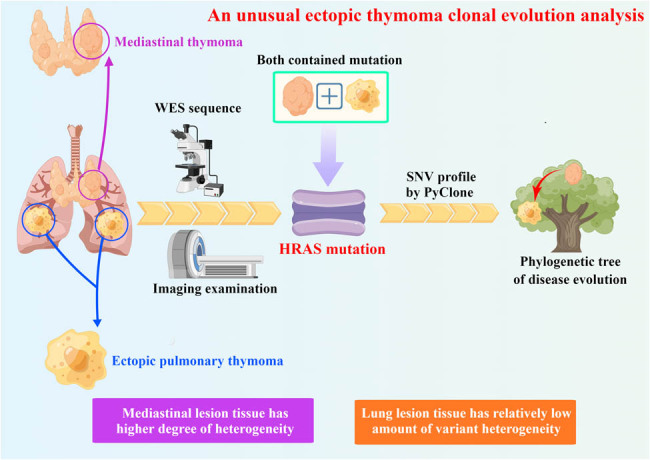

This result revealed genetic differences between type A ectopic pulmonary thymoma and mediastinal thymoma with high tumor heterogeneity.

## 1 Introduction

Thymoma is the most common anterior mediastinum tumor, while the incidence of ectopic thymoma is very low, accounting for only 4% of all thymomas [1,2]. The most common locations of ectopic thymoma are the cervical region followed by the lungs and pleura [2]. Other locations also have been reported, such as thyroid gland, middle/posterior mediastinum and pericardium [3]. Intratumoral heterogeneity hampers successful treatment in many types of solid tumors, and its impact on the course and treatment efficacy of thymomas is still an unmet scientific and clinical need [4]. The clonal architecture of thymoma plays a critical role in its pathogenesis and invasiveness [4]. Therefore, gaining more information about the genetic changes associated with thymomas could contribute to facilitate improvements in the prevention and treatment of this disease.

In this study, we reported a rare case of type A mediastinal thymoma and ectopic pulmonary thymoma. We performed whole exome sequencing (WES) of the 2 ectopic thymoma nodules in the ectopic thymoma patient and revealed a set of cancer-related genes that are recurrently mutated in thymomas, including *HRAS*. In addition, we evaluated the intratumor heterogeneity of non-silent mutations by PyClone, and clonal evolution analysis showed that these two lesions originated from multi-ancestral regions.

## 2 Methods

### 2.1 Bioinformatic analysis

The sequencing data were first tested for quality control analysis using FastQC. Next the data were aligned to the human reference genome (NCBI build 37) using BWA [5], and then sorted followed by PCR duplication removal using GATK 4.0 [6].

Somatic mutation calling was performed using Mutect2, VarDict, and Strelka2 [7]. Somatic mutations existing in at least two of the results of the three software were selected as high confident mutations. The variant data were annotated using ANNOVAR [8] and converted to MAF files using maftools for further analysis [9]. The copy number variation was analyzed by GATK4.0 with default parameters following the tutorials of Broad Institute (Sensitively detect copy ratio alterations and allelic segments, https://software.broadinstitute.org/gatk/documentation/topic?name=tutorials). We used the MapScape R package to analyze the interactive visualization of spatial clonal evolution of tumor tissue (https://bioconductor.org/packages/release/bioc/html/mapscape.html).

### 2.2 Case presentation

**Figure 1:**
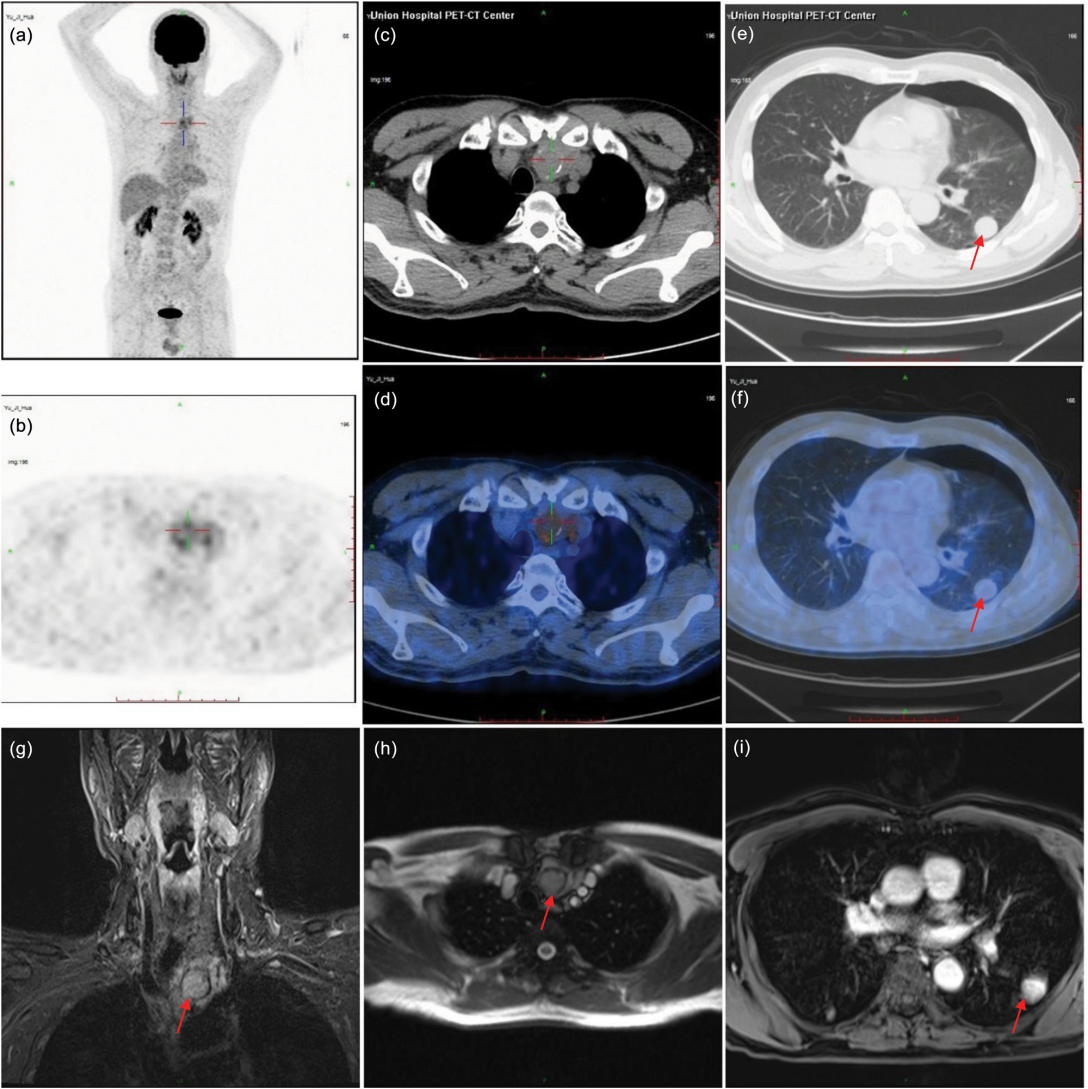
Imaging findings of the patient. (a–d) PET/CT showed a mass in the mediastinal portion. (e and f) PET/CT showed nodules (red arrow) in lung. (g and h) MRI examination showed a 4.5 cm × 3.0 cm × 3.8 cm mass (red arrow) in the mediastinal portion. (i) MRI examination showed multiple nodules in the lung. The red arrow indicated a 1.9 cm × 1.8 cm nodule in the left lower lobe of the lung.

**Figure 2:**
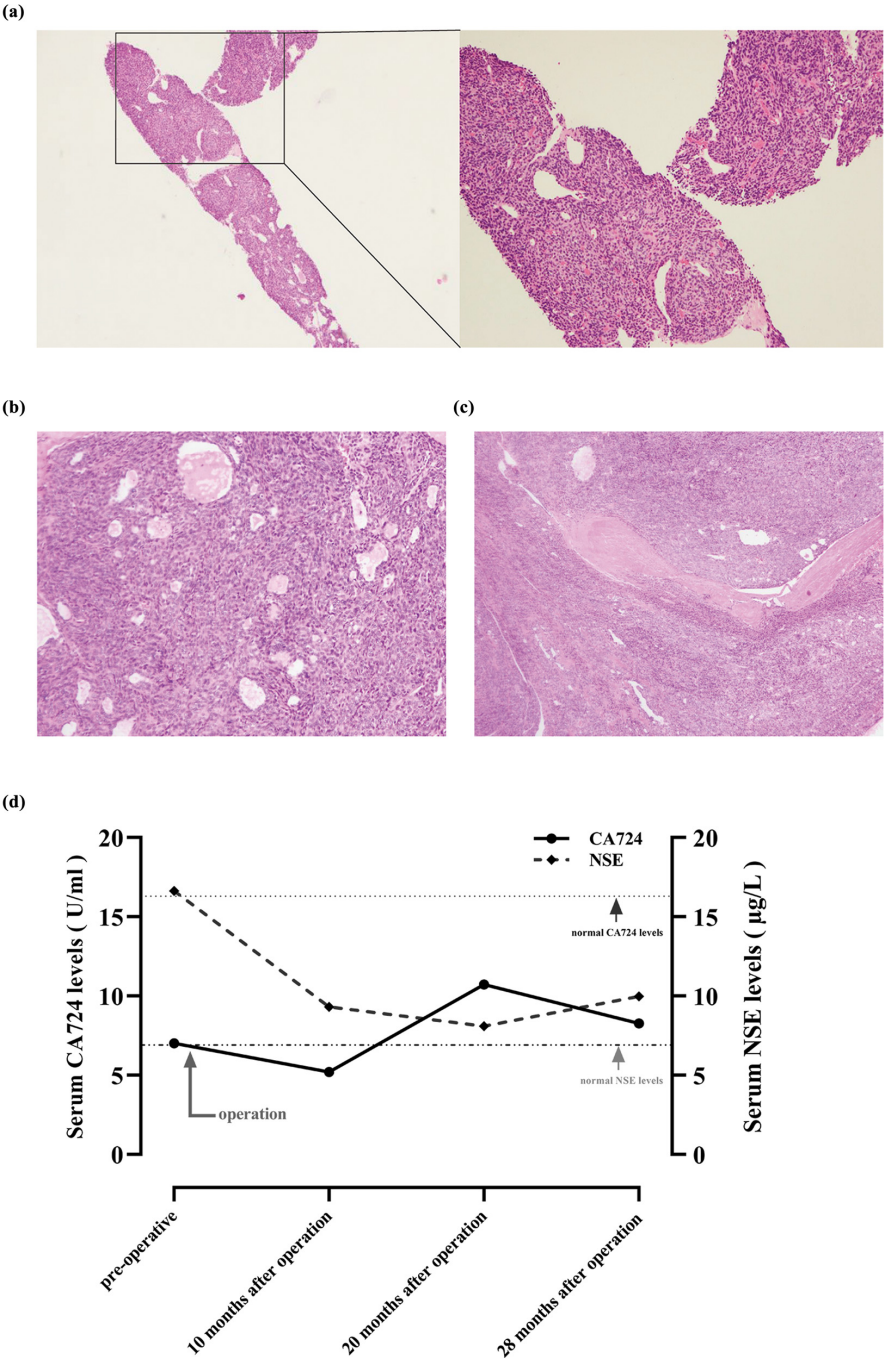
(a) Histopathological result showed a type A thymoma pattern (hematoxylin and eosin staining; magnification: ×100). (b) Microscopic view of thymoma type A in the anterior mediastinum. (c) Microscopic view of ectopic pulmonary thymomas type A in the left lower lung. Magnification: ×40. (d) Serum CA724 and NSE expression levels in patients (normal level of CA724 is less than 6.9 U/mL and the normal value of NSE is less than 16.3 μg/L).

A 62-year-old male patient was admitted to our hospital with “dizziness, nausea, and vomiting” in December 2018. Blood routine examination, liver and kidney function, electrolyte, myocardial enzyme profile, thyroid function, lymphocyte subsets, cytokines, pre-transfusion examination, tumor markers, and inflammatory indicators were performed at the initial diagnosis. No obvious abnormality was found in the physical examination. Positron emission tomography (PET) and computed tomography (CT) indicated that the patient had soft-tissue density lesions with calcification in the left lower lobes of the thymus (Figure 1a–d) and multiple nodules of different sizes in bilateral lungs (Figure 1e and f). Magnetic resonance imaging (MRI) showed the status of the mediastinum and lungs: (1) A mass (45 mm × 30 mm × 38 mm) was found in the superior mediastinum of the left lobe of thymus and fat space between the lesion and the surrounding mediastinum vascular, trachea, esophagus, and upper thymus gland; trachea shifted to the right, and the tumor showed equal T1 and long T2 mixed signals with obvious heterogeneous enhancement (Figure 1g and h); (2) Multiple nodules with smooth margin were found in bilateral lungs. The largest one (19 mm × 18 mm) was located in the left lower lobe of lung, and displayed marked enhancement (Figure 1i).

**Figure 3:**
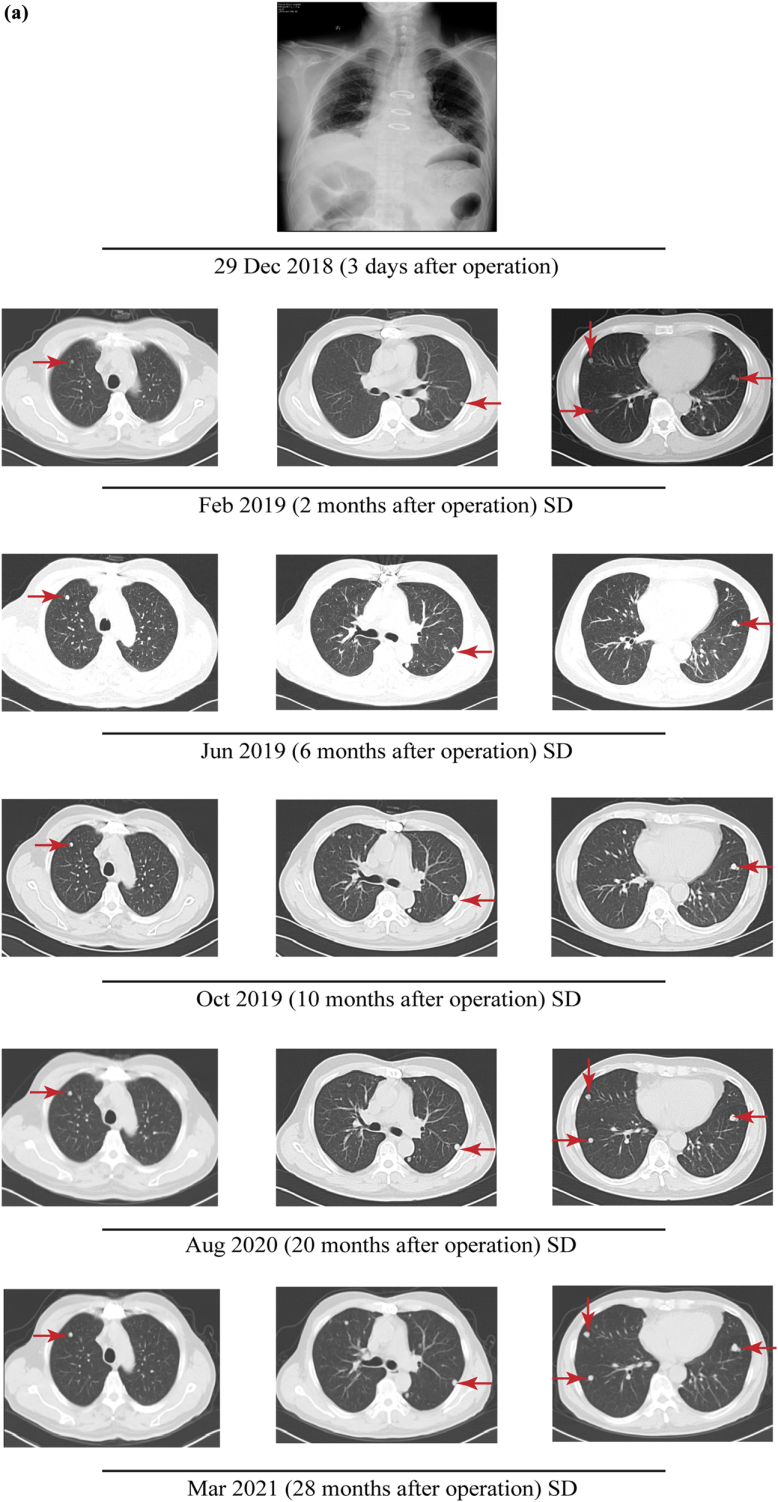
Imaging findings of the patient. CT examination showed masses (red arrows) in the multiple nodules in the lung.

According to the proportion of positive cells and the degree of staining, the immunohistochemical results were divided into five grades of “+ (the proportion of positive cells and the degree of staining >60%),” “Partial + (the proportion of positive cells and the degree of staining is about 50%),” “Local + (the proportion of positive cells and the degree of staining is within the interval of 10–50%),” “Few + (the proportion of positive cells and the degree of staining <10%),” and “– (negative).” Immunohistochemical results (lung puncture biopsy under the guidance of CT in the left lung) showed that the expression of phosphoenolpyruvate carboxykinase (PCK) and P40 in tumor cells revealed positive and the expression of CD5, CD20, CD117, terminal deoxynucleotidyl transferase (TdT), and Ki-67 revealed negative as shown in Table 1. The above immunohistochemical indexes were not detected in the tissue specimens of the ectopic thymoma of left lower lung. Similarly, for the immunohistochemical indexes thyroid transcription factor 1 (TTF-1), synapsin (Syn), CD34, signal transducer and activator of transcription 6 (STAT6), smooth muscle actin (SMA), S100, Desmin, and anaplastic lymphoma kinase (ALK) were detected in ectopic thymoma of the left lower lung but not detected in the tissue specimens of the anterior mediastinal thymoma. The anterior mediastinal nodal tissue in this patient measured 5 cm × 4 cm × 3.5 cm. The pathological manifestations showed a grayish-white section, lobulated, with the envelope still intact and an additional 4 cm × 3 cm × 1.5 cm accumulation of fat-like tissue. Further detailed review of the pathological pictures of the pulmonary nodules revealed that the pulmonary nodules contained a small amount of adipose tissue. In contrast, ectopic thymomas are composed of spindle cells, epithelial cells, and mature adipocytes. The above evidence suggests the occurrence of ectopic thymus tissue, and further provides novel points and reference value for clinical practice. Overall, further pathological results revealed the possibility of type A thymoma (Figure 2a), considering thymoma involving lung or ectopic pulmonary in the lung. At the end of December 2018, the patient underwent “thoracotomy exploration plus mediastinal lesion resection plus thoracoscopic wedge pneumonectomy” under general anesthesia. The surgeon removed the mediastinal mass and wedge resection of the lung lesion. But only one lesion of the lung was removed for cloning analysis and sequencing. During the operation, a hard mass of 3.0 cm × 3.0 cm was found in the anterior mediastinum, which was closely related to the innominate vein and the left common carotid artery, involving the left mediastinal pleura, and multiple tough nodules were seen in the left lower lobe, the larger of which was about 2.0 cm × 1.5 cm. Postoperative pathology indicated type A anterior mediastinal thymoma (Figure 2b). Multiple type A thymoma presented in the lower left lung with ectopic thymoma in the lung (Figure 2c). The patient complained of chest discomfort occasionally after operation without obvious fever, cough, chest tightness, palpitation, and other discomfort. The patient underwent surgery only and did not receive radiotherapy or chemotherapy after operation. The pulmonary lesions of the patients have been stable (Figure 3). The CA724 and NSE of the patients were high before operation, but the NSE decreased to normal after operation, and the CA724 maintained a slightly higher level until now (Figure 2d). Thereafter, no recurrence or metastasis was found after the operation and the disease remained stable for approximately 3 years under observation. The patient is still being followed up.

**Table 1:** Results of immunohistochemistry on the tumor tissues originating from lung and anterior mediastinum

Immunohistochemistry (ectopic thymoma of the left lower lung)	
P40 (positive nucleus)	Positive (>60%)
PCK (positive cytoplasm)	Positive (>60%)
TTF-1, Syn, CD34, STAT6, SMA, S100, Desmin, ALK	Negative (–)

Immunohistochemistry (anterior mediastinal thymoma)	
P40 (positive nucleus)	Positive (>60%)
PCK (positive cytoplasm)	Positive (>60%)
CD5, CD20, CD 117, TdT	Negative (–)
Ki-67	Positive (+), (5%)

To obtain insights into the genetic alterations that characterize the tumor heterogeneity of thymomas, we performed WES analysis of mediastinal lesion and lung lesion tissues and a paired peripheral blood samples from the patient. We identified 383 and 269 somatic non-synonymous single-nucleotide variations (SNVs) in the coding regions of mediastinal lesion and lung lesion tissues, respectively (Tables S1 and S2). Direct comparison between the mediastinal lesion and lung lesion tissues revealed overlapping frequencies of commonly mutated genes, a total of eight somatic mutant genes were shared (Table S3). Consistent with a previous exome sequencing analysis of thymoma and thymic Carcinoma [10,11,12], *HRAS* mutation was observed in both mediastinal lesion and lung lesion tissues. We compared results from ten canonical signaling pathways and cancer driver genes with frequent genetic alterations in our study, using recently published mutational profiles of multiple samples from TCGA publications [13], 18 genes (18/335) and 16 genes (16/299) were identified in ectopic pulmonary thymoma, respectively. 24 genes (24/335) and 26 genes (26/299) were tested in mediastinal thymoma, respectively. Additionally, compared to the DNA Repair and DNA Damage Signaling Pathways [14], there are 8 genes (8/229) and 17 genes (17/229) that contain mutations found in ectopic pulmonary thymoma and mediastinal thymoma, respectively.

**Figure 4:**
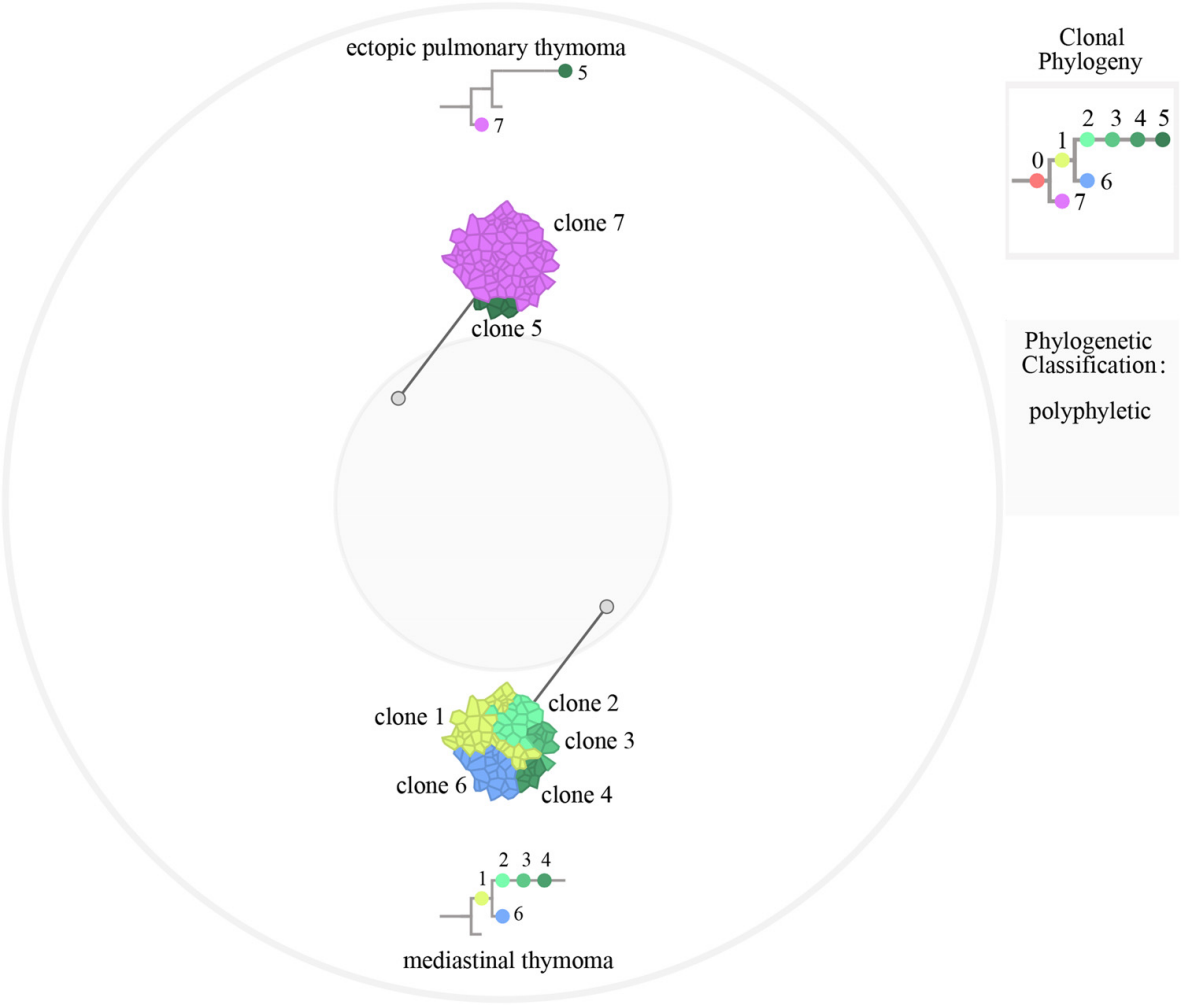
Clone phylogenies and sample clone mixtures. A clone phylogeny is shown for the patient, with distinct clonal genotypes color-coded. The clonal composition of each sample was depicted as a schematic tumor, with the coloring of “cells” proportional to the prevalence of each clone. Samples were arrayed in a circle, with constituent clones from the overall clone phylogeny represented in an outer circle.


To gain further insight into the accumulation of genetic alterations, we constructed phylogenetic trees of disease evolution taking all mutations in mediastinal thymoma and ectopic pulmonary thymoma (Figure 4). We used the detected SNV profile for each sample to analyze the corresponding clonal and subclonal architecture using PyClone. Clustering of mutations revealed the subclonal structure of mediastinal thymoma and ectopic pulmonary thymoma in patient harboring multiple mutations. The mediastinal thymoma exhibited a wide spectrum of modes over clonal and subclonal frequencies ranging from one to five clusters. Mutation of *PIK3CA* was clustered together in subclone-1. Mutation of *MUC17* was clustered in subclone-3. *BRCA2*, *ALK*, and *PIK3R1* mutations were clustered together in subclone-6. Driver gene mutation was not found in subclone-2 and subclone-4. Two clusters in ectopic pulmonary thymoma were detected by PyClone. Mutation of *MTOR* was clustered in subclone-5, and *ZNF440* mutations were clustered together in subclone-7. The results showed that the mediastinal lesion tissue appeared to have higher degree of heterogeneity in detected variants whereas the lung lesion tissue showed a relatively low amount of variant heterogeneity.

**Informed consent:** Informed consent has been obtained from all individuals included in this study.
**Ethical approval:** The research related to human use has been complied with all the relevant national regulations, institutional policies and in accordance with the tenets of the Helsinki Declaration, and has been approved by the Ethics Committees of Union Hospital, Tongji Medical College, Huazhong University of Science and Technology.

## 3 Discussion

Thymomas and thymic carcinomas (TCs) are rare and primary tumors of the mediastinum, which is derived from the thymic epithelium [15]. Thymomas are the most common primary anterior mediastinal tumor, while ectopic thymomas are very rare [16]. As a subtype of ectopic thymoma, ectopic pulmonary thymoma accounts for 20% [17], which was first described by McBurney et al. in 1951. Until 2017, only 37 cases of ectopic pulmonary thymoma have been reported [18]. Although the histogenesis and specific mechanisms of ectopic thymomas are unknown, the most widely accepted concept to explain the presence of such tumors remains the displacement and stem cell theories [19]. Pharyngeal pouches 3 and 4 contribute to the formation of the thymus [20]. A sacculation of the ventral, endodermally lined portion of the third pharyngeal pouch occurs in the sixth week of pregnancy [20]. Furthermore, cervical sinuses are attached to primordia surrounding them by ectodermally derived tissue [20]. During the eighth week, after caudal and medial migration, the thymic primordia expand toward their lower poles to form two epithelial bars that fuse in the midline [20,21]. This final descent usually leads to the tail portion of the organ becoming thin and breaking up into small fragments [21,22]. Some of these thymic remains, however, may persist and grow into ectopic thymic tissue or even TCs [23]. Embryonic tissue displacement is generally responsible for such tumors. These tumors are typically caused by the displacement of thymic tissue during embryogenesis.

In addition, this concept readily explains thyroid, paratracheal, and pericardial thymic tissue, there is no explanation for the presence of thymic rests or neoplasms in the lung or pleura. As the larynx and pharynx develop, the pulmonary system is much more advanced than the thymus [24]. Before the thymic primordium develops, it descends caudally during the fourth week of fetal life [21]. The development of peripheral intrapulmonary thymomas cannot be explained by the dispersion of thymic tissue in the developing lung. The imaging studies in this patient showed multiple lung lesions, and we need to consider the possibility that the type A thymoma metastasized. In our case, the patient was diagnosed as ectopic pulmonary thymoma and type A mediastinal thymoma. By applying the stem cell theory, it made sense that the patient in our report developed thymomas at both sites.

Although the cytologic features of ectopic thymomas are identical to those of mediastinal thymomas, the accurate diagnosis is extremely challenging due to the rarity and diversity of histology patterns found in such unusual location. Diagnosis becomes more difficult because of the lack of typical features of the thymoma or thymic carcinoma, which affects pathologists’ understanding of this entity. Ectopic pulmonary thymoma is identical to the characteristic histological features of mediastinal thymoma based on the definitive diagnosis of histologic findings [25]. However, it is easily confused with other tumors, so detection of several immunohistochemical markers such as p40, TdT, SMA, S100, CD5, and desmin is recommended [26]. The strong collagen IV deposits among the spindle cells and the extensive co-expression of cytokeratins and CD20 are considered highly diagnostic for type A thymoma according to the World Class Organization classification system of thymic epithelial tumor (TET) [27]. The hemangiopericytoma pattern is a common feature of spindle cell (type A) thymoma [27]. In addition, the diagnostic value of the co-expression of cytokeratins and CD20 was considered [27]. In our case, positive staining for p40 and PCK were observed in both tumor tissues, while other immunohistochemical markers were negative. These results together with the histological features revealed by hematoxylin and eosin stain help in the diagnosis of ectopic pulmonary thymoma and type A mediastinal thymoma according to the 2021 version of the World Health Organization classification of Tumor Series, 5th edition [28]. The recommended treatment for thymoma is surgical resection because of the significantly better survival rate [24]. Besides, adjuvant therapy such as radiation or chemotherapy is suggested in case of incomplete resection or expansion of the tumor tissue. In our case, the masses were low-risk thymomas without invasion, therefore no adjuvant therapy was conducted after the surgical treatment.

Thymomas rarely metastasize, whereas TCs exhibit a more aggressive behavior, with distant metastases in liver, bones, or lymph nodes, and are frequently symptomatic due to the extent of local invasion. Type A thymoma is usually characterized by a low grade of malignancy, and we found genetic differences between mediastinal thymoma (type A) and ectopic pulmonary thymoma in our case. A total of eight genes were found to be co-mutated in both mediastinal lesion and lung lesion tissues, and *HRAS* mutation was identified as one of the recurrent somatic mutations in thymic carcinoma, which is the most aggressive type of TCs [29]. Briefly, the diagnosis of type A mediastinal thymoma and ectopic pulmonary thymoma was further validated from the point of view of clonal and mutational heterogeneity. Petrini et al. [30] analyzed 28 TETs and identified a high frequency of *GTF2I* (general transcription factor II I) mutation in the type A thymoma but not in the aggressive subtypes. *GTF2I* mutation was not found in either ectopic pulmonary thymoma or mediastinal thymoma. To further understand the function of the identified gene mutations, we reviewed the literature reports addressing these mutations and found that most of these mutations have not been reported to be related to human tumors at present [29]. Spatial heterogeneity complicates the analysis of solid tumors, as different areas of the tumor may contain different subclonal populations [31]. Analysis of multiple regions of heterogeneous tumors contributes to reveal the full spectrum of mutations in tumors and to identify the spatial origin of subclones. By sequencing the mediastinal lesion and lung lesion tissues, we elucidated the extent of genomic heterogeneity and the evolutionary history of ectopic pulmonary thymoma and type A mediastinal thymoma. We constructed a phylogenetic tree of disease evolution using all mutations in mediastinal thymoma and ectopic pulmonary thymoma as shown in Figure 4. Two clusters of ectopic pulmonary thymoma and mediastinal thymoma were detected by PyClone, and they showed a wide spectrum of modes over clonal/subclonal frequencies ranging from one to five clusters. In this patient, both the mediastinal lesion and lung lesion tissues were polyclonal, and the clonal populations differed from one thymoma to another, the mediastinal lesion tissue had a high degree of heterogeneity and the lung lesion tissue showed a relatively low amount of variant heterogeneity. No other lesions or ectopic thymus were found by fluorodeoxyglucose-positron emission tomography or whole-body computerized tomography (CT) scan, which assisted to confirm the multi-ancestral regions of the disease. All in all, the novel insights of the study are the following two points. First, the mediastinal lesion tissue has higher degree of heterogeneity and the lung lesion tissue has relatively low amount of variant heterogeneity in the detected variants. Second, clonal evolution analysis showed that these two lesions originated from multi-ancestral regions.

The following are the implications and clinical value of the study for future research. The results of the genetic analysis in this study suggest that the two lesions, type A mediastinal thymoma and ectopic pulmonary thymoma, may be derived from multi-ancestral regions, laying the foundation for the polyclonal evolution of the disease. In addition, this study reveals that IHC staining of spindle cells, epithelial cells, and mature adipocytes can suggest the occurrence of ectopic thymus tissue, which provides a new perspective and reference value for clinical practice. Moreover, HRAS, as a commonly mutated gene, could be a potential therapeutic target in the future due to its gene function and high mutation rate in thymoma. More importantly, this study illustrates the pivotal role of genetic analysis and molecularly targeted therapy in the identification and treatment of ectopic thymoma.

This study has several limitations. First, postoperative therapy is limited and not standardized due to the low incidence of this disease. Complete resection, followed by chemotherapy and radiotherapy, is the generally accepted strategy. In this patient, the surgery was performed without radiotherapy or chemotherapy. To date there is no sign of disease recurrence after surgery. Second, myasthenia gravis is the most common adverse effect in patients with thymoma, and it is logical that the study should follow the patients’ hormone levels over time. Although this study was a case report, we believe the results of the analyses of large sample were convincing.

## 4 Conclusion

In conclusion, our results revealed genetic differences between type A ectopic pulmonary thymoma and mediastinal thymoma with high tumor heterogeneity.

## Supplementary Material

Supplementary Table

## References

[1] Álvarez-Velasco R, Gutiérrez-Gutiérrez G, Trujillo JC, Martínez E, Segovia S, Arribas-Velasco M, et al. Clinical characteristics and outcomes of thymoma-associated myasthenia gravis. Eur J Neurol. 2021;28(6):2083–91. 10.1111/ene.14820 33721382

[2] Moran CA. Thymoma Staging: An Analysis of the Different Schemas. Adv Anat Pathol. 2021;28(5):298–306. 10.1097/PAP.0000000000000315 34326287

[3] Ao Y-Q, Jiang J-H, Gao J, Wang H-K, Ding J-Y. Recent thymic emigrants as the bridge between thymoma and autoimmune diseases. Biochim Biophys Acta Rev Cancer. 2022;1877(3):188730. 10.1016/j.bbcan.2022.188730 35469968

[4] Liu D, Zhang P, Zhao J, Yang L, Wang W. Identification of Molecular Characteristics and New Prognostic Targets for Thymoma by Multiomics Analysis. Biomed Res Int. 2021;2021:5587441. 10.1155/2021/5587441 PMC8159640 34104648

[5] Mohideen AMSH, Johansen SD, Babiak I. High-throughput identification of adapters in single-read sequencing data. Biomolecules. 2020;10(6):878. 10.3390/biom10060878 PMC7356586 32521604

[6] Gabrielaite M, Torp MH, Rasmussen MS, Andreu-Sánchez S, Vieira FG, Pedersen CB, et al. A comparison of tools for copy-number variation detection in germline whole exome and whole genome sequencing data. Cancers (Basel). 2021;13(24):6283. 10.3390/cancers13246283 PMC8699073 34944901

[7] Chen Z, Yuan Y, Chen X, Chen J, Lin S, Li X, et al. Systematic comparison of somatic variant calling performance among different sequencing depth and mutation frequency. Sci Rep. 2020;10(1):3501. 10.1038/s41598-020-60559-5 PMC7044309 32103116

[8] Liu J, Cao Y, Zhu K, Yao S, Yuan M, Kong X, et al. Early evaluation of subclinical cardiotoxicity in patients with lung cancer receiving immune checkpoint inhibitors by cardiovascular magnetic resonance: a prospective observational study. Quant imaging Med Surg. 2022;12(10):4771–85. 10.21037/qims-22-41 PMC9511417 36185042

[9] Ferrer-Bonsoms JA, Jareno L, Rubio A. Rediscover: an R package to identify mutually exclusive mutations. Bioinformatics. 2022;38(3):844–5. 10.1093/bioinformatics/btab709 34664620

[10] Hou X, Lin S, Liu Y, Wang K, Yu Z, Jia J, et al. Analysis of the tumor microenvironment and mutation burden identifies prognostic features in thymic epithelial tumors. Am J Cancer Res. 2022;12(5):2387–96. PMC9185609 35693087

[11] Nakamura Y, Sato H, Miyano Y, Murakami R, Motoki M, Shigekiyo T, et al. Whole-exome sequencing and human leukocyte antigen analysis in familial myasthenia gravis with thymoma: Case report and literature review. Clin Neurol Neurosurg. 2021;208:106864. 10.1016/j.clineuro.2021.106864 34388596

[12] Yu X-T, Yu L, Du X, Yu Z, Yang X-G, Jiang Y-X. Clinical and genetic characteristics of thymoma patients with autoimmune hepatitis and myocarditis. Front Oncol. 2021;11:746304. 10.3389/fonc.2021.746304 PMC8777214 35070964

[13] Ke X, Wu H, Chen Y-X, Guo Y, Yao S, Guo M-R, et al. Individualized pathway activity algorithm identifies oncogenic pathways in pan-cancer analysis. EBioMedicine. 2022;79:104014. 10.1016/j.ebiom.2022.104014 PMC9117264 35487057

[14] Huang R-X, Zhou P-K. DNA damage response signaling pathways and targets for radiotherapy sensitization in cancer. Signal Transduct Target Ther. 2020;5(1):60. 10.1038/s41392-020-0150-x PMC7192953 32355263

[15] Muto Y, Okuma Y. Therapeutic options in thymomas and thymic carcinomas. Expert Rev Anticancer Ther. 2022;22(4):401–13. 10.1080/14737140.2022.2052278 35266421

[16] Yasumizu Y, Ohkura N, Murata H, Kinoshita M, Funaki S, Nojima S, et al. Myasthenia gravis-specific aberrant neuromuscular gene expression by medullary thymic epithelial cells in thymoma. Nat Commun. 2022;13(1):4230. 10.1038/s41467-022-31951-8 PMC9305039 35869073

[17] Weissferdt A, Moran CA. The spectrum of ectopic thymomas. Virchows Arch. 2016;469(3):245–54. 10.1007/s00428-016-1967-0 27255665

[18] Rückert J-C, Elsner A, Andreas MN. Mediastinal tumors. Zentralbl Chir. 2022;147(1). 10.1055/a-1674-0693 35235970

[19] Zu Y, Luo Y, Li C, Zhao J, He T, Shi X, et al. Complete remission following icotinib administration in an advanced ectopic thymic carcinoma patient harbouring the EGFR exon 19 deletion. J Gene Med. 2021;23(7):e3340. 10.1002/jgm.3340 PMC8365660 33835620

[20] Miller JFAP. The function of the thymus and its impact on modern medicine. Science. 2020;369(6503):eaba2429. 10.1126/science.aba2429 32732394

[21] Figueiredo M, Zilhão R, Neves H. Thymus inception: molecular network in the early stages of thymus organogenesis. Int J Mol Sci. 2020;21(16):5765. 10.3390/ijms21165765 PMC7460828 32796710

[22] Wee T, Lee AF, Nadel H, Bray H. The paediatric thymus: recognising normal and ectopic thymic tissue. Clin Radiol. 2021;76(7):477–87. 10.1016/j.crad.2021.02.017 33762135

[23] James KD, Jenkinson WE, Anderson G. Non-epithelial stromal cells in thymus development and function. Front Immunol. 2021;12:634367. 10.3389/fimmu.2021.634367 PMC7946857 33717173

[24] Marx A, Chan JKC, Chalabreysse L, Dacic S, Detterbeck F, French CA, et al. The 2021 WHO classification of tumors of the thymus and mediastinum: what is new in thymic epithelial, germ cell, and mesenchymal tumors? J Thorac Oncol. 2022;17(2):200–13. 10.1016/j.jtho.2021.10.010 34695605

[25] Tatematsu T, Okuda K, Endo K, Hattori H, Matsui T, Oda R, et al. Type A thymoma with simultaneous solitary intrapulmonary metastasis: A case report. Thorac Cancer. 2021;12(12):1923–6. 10.1111/1759-7714.13975 PMC8201543 33960662

[26] Krassas A, Diamantis I, Karampinis I, Vgenopoulou S, Misthos P. Primary intrapulmonary thymoma appearing as a solitary pulmonary nodule: The “Master of Disguise” of Lung Tumors?: Case Report. J Chest Surg. 2021;54(5):412–5. 10.5090/jcs.20.116 PMC8548194 33293484

[27] Shin DW, Cho JH, Ha J, Jung K-W. Trends in incidence and survival of patients with thymic epithelial tumor in a high-incidence Asian country: analysis of the Korean central cancer registry 1999 to 2017. J Thorac Oncol. 2022;17(6):827–37. 10.1016/j.jtho.2022.02.001 35158083

[28] Zhou Q, Huang X, Xue C, Zhou J. Correlation of clinical and computed tomography features of thymic epithelial tumours with World Health Organization classification and Masaoka-Koga staging. Eur J Cardiothorac Surg. 2022;61(4):742–8. 10.1093/ejcts/ezab349 34329409

[29] Kuhn E, Pescia C, Mendogni P, Nosotti M, Ferrero S. Thymic epithelial tumors: An evolving field. Life (Basel). 2023;13(2):314. 10.3390/life13020314 PMC9964105 36836670

[30] Petrini I, Meltzer PS, Kim I-K, Lucchi M, Park K-S, Fontanini G, et al. A specific missense mutation in GTF2I occurs at high frequency in thymic epithelial tumors. Nat Genet. 2014;46(8):844–9. 10.1038/ng.3016 PMC5705185 24974848

[31] Miller CA, White BS, Dees ND, Griffith M, Welch JS, Griffith OL, et al. SciClone: inferring clonal architecture and tracking the spatial and temporal patterns of tumor evolution. PLoS Comput Biol. 2014;10(8):e1003665. 10.1371/journal.pcbi.1003665 PMC4125065 25102416

