# Supplementary material for: An unusual ectopic thymoma clonal evolution analysis: A case report
Source: Open Life Sci. 2023 May 18;18(1):20220600. doi: 10.1515/biol-2022-0600 (PMC10199323; doi:10.1515/biol-2022-0600)
Supplement: Supplementary Table [file biol-2022-0600-sm.pdf]

Supplementary material

Table S1: The specific somatic mutations found in mediastinal lesion

Gene	Chr		Sequence			
		Position	Reference_Allele	Tumor_Seq_Allele1	Tumor_Seq_Allele2	HGVSp_Short
TMEM52	chr1	1849419	T	T	C	p.S178G
DFFB	chr1	3774202	C	C	A	p.A12D
CHD5	chr1	6214830	G	G	A	p.A212V
UBE4B	chr1	10211469	C	C	T	p.R926*
CASZ1	chr1	10699940	C	C	T	p.A1447T
EFHD2	chr1	15736580	C	C	G	p.A38G
CROCC	chr1	17256398	C	C	G	p.L137V
ATP13A2	chr1	17318761	G	G	A	p.A661V
RCC2	chr1	17764982	GCC	GCC	—	p.A10del
ARHGEF10L	chr1	17982532	C	C	G	p.S880R
LDLRAD2	chr1	22141295	G	G	A	p.E164K
MYOM3	chr1	24389648	G	G	T	p.N1246K
NR0B2	chr1	27240268	G	G	A	p.T55I
ZSCAN20	chr1	33957137	C	C	A	p.L427I
TEKT2	chr1	36553389	C	C	A	p.Q354K
COL8A2	chr1	36563903	C	C	T	p.G460D
HPCAL4	chr1	40148335	C	C	T	p.R150H
SLC6A9	chr1	44477265	G	G	A	p.L73F
LRRC41	chr1	46746985	C	C	T	p.R523H
KANK4	chr1	62739883	A	A	G	p.L298P
RPE65	chr1	68912499	G	G	C	p.P47A
SPAG17	chr1	118539321	A	A	T	p.L1608M
BCL9	chr1	147092198	A	A	G	p.E746G
TCHHL1	chr1	152058983	T	T	C	p.K392R
INSRR	chr1	156821756	G	G	A	p.R289C
IGSF9	chr1	159901275	C	C	T	p.R494Q
CREG1	chr1	167522800	C	C	T	p.R60H
FMO1	chr1	171254372	T	T	A	p.S430T
ASPM	chr1	197071297	C	C	T	p.V2362I
ASPM	chr1	197112403	C	C	T	p.G327R
CNTN2	chr1	205030533	C	C	A	p.R320S
OBSCN	chr1	228461987	G	G	A	p.R2217H
OBSCN	chr1	228470742	C	C	T	p.P3261S

(*Continued*)

Table S1: *Continued*

Gene	Chr		Sequence			
		Position	Reference_Allele	Tumor_Seq_Allele1	Tumor_Seq_Allele2	HGVSp_Short
TAF5L	chr1	229750217	G	G	A	p.R5C
ZP4	chr1	238050768	G	G	A	p.A216V
SMYD3	chr1	246091247	C	C	T	p.E230K
CNST	chr1	246797306	C	C	A	p.Q233K
OR6F1	chr1	247875319	C	C	T	p.V247M
KLHL29	chr2	23918686	C	C	A	p.S579Y
KLHL29	chr2	23926168	G	G	A	p.E740K
TMEM214	chr2	27255993	C	C	A	p.A37E
IFT172	chr2	27672866	G	G	T	p.A1350E
ALK	chr2	29416517	G	G	A	p.A1479V
TMEM178A	chr2	39893321	C	C	A	p.D69E
LRPPRC	chr2	44116950	A	A	T	p.L1351M
PSME4	chr2	54114578	T	T	C	p.D1516G
REL	chr2	61149202	C	C	A	p.N464K
SLC1A4	chr2	65216871	G	G	A	p.A32T
PCYOX1	chr2	70488424	T	T	C	p.F134L
EGR4	chr2	73518748	G	G	A	p.T536I
SLC4A5	chr2	74459600	C	C	T	p.V924I
MOGS	chr2	74692308	C	C	T	p.A23T
SEMA4F	chr2	74900869	G	G	A	p.E246K
KRCC1	chr2	88327923	G	G	T	p.P54T
VWA3B	chr2	98928475	G	G	T	p.G1239W
GPR39	chr2	133402935	G	G	A	p.R373H
ACVR1	chr2	158617544	C	C	T	p.G371E
SCN3A	chr2	165970430	C	C	A	p.G1189W
SCN3A	chr2	165987786	C	C	T	p.E845K
SCN1A	chr2	166901794	C	C	G	p.S474T
DLX2	chr2	172965494	G	G	T	p.P255Q
ORMDL1	chr2	190640307	T	T	C	p.I104V
SATB2	chr2	200137337	G	G	A	p.A600V
4-Mar	chr2	217234887	G	G	A	p.R33C
CHPF	chr2	220406787	G	G	T	p.R147S
TMEM198	chr2	220414002	C	C	T	p.P291S
STK11IP	chr2	220476713	G	G	A	p.R802Q
PID1	chr2	229890448	G	G	T	p.A216D
ANKMY1	chr2	241465660	G	G	A	p.L297F
PPP1R7	chr2	242122188	C	C	T	p.L345F
FANCD2	chr3	10138031	A	A	T	p.T1354S
FGD5	chr3	14860961	C	C	A	p.A128E

(*Continued*)

Table S1: *Continued*

Gene	Chr		Sequence			
		Position	Reference_Allele	Tumor_Seq_Allele1	Tumor_Seq_Allele2	HGVSp_Short
SCN5A	chr3	38648178	C	C	T	p.W374*
CSRNP1	chr3	39184858	G	G	T	p.S486R
SNRK	chr3	43389478	G	G	A	p.S576N
ALS2CL	chr3	46713506	C	C	T	p.R851Q
ALS2CL	chr3	46716057	T	T	C	p.K810E
SLC26A6	chr3	48668143	G	G	A	p.A382V
CELSR3	chr3	48689349	G	G	A	p.R1962W
LAMB2	chr3	49163234	G	G	A	p.R812C
TMEM115	chr3	50392805	A	A	G	p.I342T
STAB1	chr3	52557503	G	G	A	p.A2401T
RP11-159G9.5	chr3	88108596	G	G	A	p.G43S
MCM2	chr3	127335845	G	G	C	p.A553P
ACAD9	chr3	128625073	G	G	A	p.R420H
MME	chr3	154832942	A	A	G	p.K119R
SI	chr3	164741401	C	C	G	p.R1019P
PIK3CA	chr3	178952085	A	A	G	p.H1047R
MFI2	chr3	196751336	C	C	T	p.A109T
FAM193A	chr4	2701917	C	C	A	p.L1049M
CRMP1	chr4	5843118	G	G	A	p.A357V
CCDC96	chr4	7043648	G	G	T	p.Q340K
AFAP1	chr4	7840267	G	G	T	p.A237D
EPHA5	chr4	66356221	C	C	A	p.V426F
CFI	chr4	110687775	TGTTGACAG	TGTTGACAG	—	p.Y85_Q88delins*
FAT4	chr4	126355498	A	A	G	p.I2373V
DCLK2	chr4	151000540	C	C	A	p.R121S
CENPU	chr4	185621975	G	G	A	p.R329W
NIPBL	chr5	36976334	C	C	A	p.S442*
MROH2B	chr5	41039557	C	C	T	p.R685Q
PIK3R1	chr5	67593247	G	G	T	p.G665C
ENC1	chr5	73932031	G	G	T	p.H94N
GPR98	chr5	90136714	T	T	C	p.L5644P
APC	chr5	112164644	T	T	C	p.M573T
ZNF608	chr5	123984353	G	G	T	p.A575D
FBN2	chr5	127627319	T	T	C	p.N2065S
SOWAHA	chr5	132150344	T	T	A	p.M344K
SLC23A1	chr5	138713693	A	A	G	p.L479P
PCDHA2	chr5	140176333	G	G	A	p.R595H
PCDHA5	chr5	140201458	C	C	A	p.S33*
PCDHA5	chr5	140203285	C	C	A	p.P642Q

(*Continued*)

Table S1: *Continued*

Gene	Chr		Sequence			
		Position	Reference_Allele	Tumor_Seq_Allele1	Tumor_Seq_Allele2	HGVSp_Short
PCDHB16	chr5	140564305	G	G	A	p.R724H
EFCAB9	chr5	171626528	C	C	T	p.A93V
CDHR2	chr5	176002812	C	C	T	p.A311V
RIPK1	chr6	3083440	C	C	A	p.A194E
SLC17A3	chr6	25862526	A	A	G	p.S80P
GPX6	chr6	28472223	C	C	A	p.W171L
FGD2	chr6	36978804	C	C	A	p.A119E
CUL9	chr6	43164394	C	C	T	p.A866V
POLH	chr6	43572374	C	C	T	p.R303*
CAPN11	chr6	44137260	C	C	T	p.R111W
GPR110	chr6	46967985	G	G	T	p.L903I
ENPP1	chr6	132206176	G	G	A	p.R806H
SHPRH	chr6	146264395	C	C	T	p.V708I
LATS1	chr6	150023246	T	T	C	p.K6R
SLC22A3	chr6	160858034	C	C	A	p.T360K
ENTPD4	chr8	23299561	C	C	T	p.G229D
SLC25A37	chr8	23429262	C	C	A	p.A304E
C8orf88	chr8	91990808	C	C	T	p.R49K
SYBU	chr8	110587284	C	C	T	p.A615T
SLC30A8	chr8	118183399	T	T	C	p.V319A
SNTB1	chr8	121554066	C	C	T	p.G503D
IL2RA	chr10	6063635	G	G	T	p.P130Q
OGDHL	chr10	50943375	C	C	T	p.A978T
H2AFY2	chr10	71859987	G	G	A	p.G238R
CDHR1	chr10	85974213	G	G	A	p.A806T
LRIT1	chr10	85997375	G	G	T	p.L64M
CYP2C8	chr10	96829091	G	G	T	p.S23R
CRTAC1	chr10	99770937	G	G	A	p.T61I
NKX2-3	chr10	101295087	G	G	A	p.G235D
TUBGCP2	chr10	135102384	C	C	T	p.D529N
ECHS1	chr10	135179572	T	T	C	p.E216G
B4GALNT4	chr11	379665	T	T	C	p.W818R
HRAS	chr11	534286	C	C	G	p.G13R
PHRF1	chr11	611074	G	G	T	p.V1599L
TRIM68	chr11	4621937	G	G	A	p.R343C
OR51S1	chr11	4870024	G	G	A	p.H139Y
OR52J3	chr11	5068212	C	C	A	p.R153S
DCHS1	chr11	6643120	C	C	G	p.A3263P
DCHS1	chr11	6661203	C	C	T	p.V548M

(*Continued*)

Table S1: *Continued*

Gene	Chr		Sequence			
		Position	Reference_Allele	Tumor_Seq_Allele1	Tumor_Seq_Allele2	HGVSp_Short
OR2D2	chr11	6913341	G	G	A	p.R131C
ARNTL	chr11	13397259	C	C	A	p.N424K
KCNC1	chr11	17803262	A	A	G	p.E580G
HTATIP2	chr11	20403759	T	T	A	p.F193L
CKAP5	chr11	46839861	T	T	C	p.K84R
OR4A5	chr11	51411722	T	T	C	p.Y225C
ZFP91	chr11	58384219	C	C	A	p.A378D
OR5A1	chr11	59211321	C	C	A	p.A227E
MS4A15	chr11	60540897	C	C	A	p.S146R
TEX40	chr11	64068274	G	G	A	p.G56D
SAC3D1	chr11	64808829	A	A	C	p.Q22P
SIPA1	chr11	65409825	C	C	T	p.R262C
C11orf68	chr11	65685451	T	T	C	p.I121V
DPP3	chr11	66254790	G	G	A	p.A184T
CTD-3074O7.11	chr11	66297371	G	G	A	p.S511N
ADRBK1	chr11	67049398	G	G	A	p.R311H
SSH3	chr11	67076890	G	G	A	p.A362T
ARAP1	chr11	72418325	G	G	A	p.R540C
ARAP1	chr11	72423557	G	G	T	p.S268R
FAM181B	chr11	82444639	C	C	T	p.A45T
TRIM49C	chr11	89774569	A	A	G	p.K404E
HTR3B	chr11	113802517	T	T	A	p.M99K
BCL9L	chr11	118772324	G	G	A	p.R710W
OR8D1	chr11	124179810	G	G	T	p.L285M
ROBO3	chr11	124746224	C	C	T	p.R883W
SACS	chr13	23904311	T	T	C	p.I4568M
BRCA2	chr13	32936668	G	G	T	p.C2605F
N4BP2L2	chr13	33012818	T	T	G	p.I736L
FOXO1	chr13	41134835	T	T	C	p.K265E
PCDH8	chr13	53420983	C	C	T	p.G530D
IRS2	chr13	110438349	G	G	T	p.P18T
TEP1	chr14	20850144	G	G	A	p.A1451V
CPNE6	chr14	24545428	G	G	A	p.R332Q
CTSG	chr14	25043948	G	G	A	p.A91V
NPAS3	chr14	34145499	T	T	C	p.L214P
ZFP36L1	chr14	69256852	G	G	T	p.H139N
AREL1	chr14	75142625	C	C	T	p.R286H
PTPN21	chr14	88946451	G	G	A	p.P442S
IFI27L1	chr14	94568292	G	G	A	p.G65D

(*Continued*)

Table S1: *Continued*

Gene	Chr	Position	Sequence			HGVSp_Short
			Reference_Allele	Tumor_Seq_Allele1	Tumor_Seq_Allele2	
ZFYVE21	chr14	104194182	G	G	A	p.A97T
NEURL4	chr17	7220867	T	T	C	p.T1411A
CHD3	chr17	7812540	C	C	T	p.P1884L
PFAS	chr17	8159686	C	C	T	p.S261L
PIK3R5	chr17	8791888	G	G	A	p.R406C
COX10	chr17	14110145	G	G	A	p.G316E
RAI1	chr17	17697356	C	C	A	p.P365Q
TOP3A	chr17	18194308	G	G	T	p.R439S
ALDH3A2	chr17	19559886	A	A	T	p.R227*
NLE1	chr17	33460419	A	A	G	p.L438P
GPR179	chr17	36486066	G	G	T	p.S1129*
OSBPL7	chr17	45892669	T	T	C	p.Q390R
HOXB13	chr17	46805756	G	G	T	p.P67H
NGFR	chr17	47583762	G	G	A	p.D104N
SPATA20	chr17	48631747	G	G	A	p.R698H
ABCC3	chr17	48761398	G	G	A	p.R1348H
CA10	chr17	50008405	T	T	C	p.H75R
BPTF	chr17	65942163	C	C	A	p.Q2447K
USH1G	chr17	72916522	C	C	T	p.D137N
LLGL2	chr17	73564644	A	A	T	p.Q375L
RECQL5	chr17	73625509	G	G	A	p.P665L
TNRC6C	chr17	76045583	C	C	T	p.T147I
GCGR	chr17	79768804	C	C	A	p.H89Q
MAFG	chr17	79880689	G	G	T	p.A94D
C22orf39	chr22	19435032	T	T	C	p.K58E
PRAME	chr22	22890691	A	A	G	p.V443A
MYO18B	chr22	26165342	G	G	A	p.G487R
C22orf31	chr22	29456600	G	G	T	p.L79I
RNF215	chr22	30783122	G	G	A	p.R61W
OSBP2	chr22	31283441	A	A	G	p.E379G
NPTXR	chr22	39239333	G	G	T	p.D177E
SCUBE1	chr22	43614411	C	C	T	p.A581T
CELSR1	chr22	46787562	G	G	A	p.P2039L
MLC1	chr22	50523237	G	G	A	p.A32V
NCAPH2	chr22	50956566	C	C	T	p.Q169*
TYMP	chr22	50965098	G	G	T	p.R279S
ATXN3L	chrX	13337209	A	A	C	p.I282R
MAGEB4	chrX	30261064	T	T	C	p.L271P
DMD	chrX	32715988	T	T	C	p.Q320R

(*Continued*)

Table S1: *Continued*

Gene	Chr		Sequence			
		Position	Reference_Allele	Tumor_Seq_Allele1	Tumor_Seq_Allele2	HGVSp_Short
LANCL3	chrX	37526685	T	T	C	p.V349A
PRICKLE3	chrX	49040227	T	C	C	p.E91G
GSPT2	chrX	51486957	C	C	T	p.P79S
IQSEC2	chrX	53285045	CTC	CTC	—	p.E312del
AWAT2	chrX	69263820	G	G	A	p.H75Y
CDX4	chrX	72667121	C	C	T	p.A11V
BHLHB9	chrX	102004957	G	G	A	p.R345Q
TBC1D8B	chrX	106091522	G	G	A	p.R609H
KIAA1210	chrX	118230621	A	A	G	p.S368P
RNF113A	chrX	119005494	G	G	A	p.A28V
CCDC160	chrX	133379717	A	A	G	p.N296S
ZNF449	chrX	134483180	C	C	A	p.A167E
MECP2	chrX	153363116	C	C	T	p.A3T
HOXA13	chr7	27239651	C	C	T	p.V16I
NOD1	chr7	30491117	T	T	A	p.Q639L
BMPER	chr7	34125556	T	T	C	p.F533L
SUGCT	chr7	40535973	T	T	A	p.F366L
AUTS2	chr7	70254761	C	C	A	p.S853R
STEAP4	chr7	87912364	G	G	T	p.Y192*
AZGP1	chr7	99566015	C	C	T	p.E126K
C7orf61	chr7	100054550	G	G	T	p.T149N
MUC17	chr7	100676377	A	A	G	p.I560M
MUC17	chr7	100680490	A	A	G	p.I1931M
MUC17	chr7	100682528	A	A	G	p.T2611A
MUC17	chr7	100684932	T	T	C	p.V3412A
RNF148	chr7	122342132	G	G	A	p.R225*
ARF5	chr7	127230151	G	G	A	p.R97Q
TMEM209	chr7	129821463	C	C	T	p.E374K
PODXL	chr7	131195635	C	C	T	p.A220T
EXOC4	chr7	133002132	G	G	A	p.G251R
SLC35B4	chr7	133981176	A	A	G	p.M239T
KDM7A	chr7	139876665	C	C	T	p.A25T
SHH	chr7	155599058	G	G	A	p.A165V
GLIS3	chr9	4118268	G	G	A	p.Q404*
KIAA2026	chr9	5968666	C	C	T	p.G522E
FREM1	chr9	14851391	A	A	G	p.L348P
ADAMTSL1	chr9	18721651	C	C	T	p.P665L
TEK	chr9	27209184	C	C	A	p.H881N
UNC13B	chr9	35403989	A	A	G	p.K1579E

(*Continued*)

Table S1: *Continued*

Gene	Chr		Sequence			
		Position	Reference_Allele	Tumor_Seq_Allele1	Tumor_Seq_Allele2	HGVSp_Short
ARHGEF39	chr9	35665106	G	G	A	p.R21W
FBXO10	chr9	37537663	T	T	C	p.D288G
GOLM1	chr9	88648227	C	C	T	p.A367T
SPATA31E1	chr9	90502155	G	G	A	p.G918D
OGN	chr9	95155416	G	G	A	p.R127*
RGS3	chr9	116346595	A	A	G	p.E968G
DAB2IP	chr9	124535278	C	C	A	p.A796E
SPTAN1	chr9	131379956	C	C	T	p.R1799W
NUP188	chr9	131764245	G	G	A	p.G1376D
CRAT	chr9	131860360	C	C	T	p.D466N
TSC1	chr9	135781272	T	T	C	p.S565G
SOHLH1	chr9	138588505	G	G	T	p.A205E
CAMSAP1	chr9	138713409	G	G	T	p.T1033K
TPRN	chr9	140086802	G	G	T	p.T661N
CDCA3	chr12	6959681	C	C	T	p.R67H
C3AR1	chr12	8212355	A	A	G	p.C143R
NECAP1	chr12	8242617	G	G	A	p.E61K
STK38L	chr12	27450664	C	C	T	p.T4M
PPFIBP1	chr12	27825383	C	C	T	p.A446V
DDN	chr12	49391472	C	C	T	p.R396H
KMT2D	chr12	49443541	G	G	T	p.T1277K
FAM186B	chr12	49994736	C	C	A	p.E229D
KRT77	chr12	53086585	C	C	T	p.R387H
SPRYD3	chr12	53468465	C	C	T	p.A159T
HOXC8	chr12	54403378	C	C	A	p.Q104K
DNAJC14	chr12	56215874	C	C	T	p.A666T
ANKRD52	chr12	56637616	T	T	A	p.Q978L
PRIM1	chr12	57140568	T	T	A	p.K147*
MYO1A	chr12	57430564	C	C	T	p.G756R
AGAP2	chr12	58131858	C	C	G	p.A58P
GLT8D2	chr12	104396938	G	G	A	p.L87F
APPL2	chr12	105600952	C	C	T	p.A176T
CCDC63	chr12	111319091	A	A	G	p.R282G
MAPKAPK5	chr12	112303121	C	C	T	p.P35L
HECTD4	chr12	112622815	G	G	A	p.R3173W
HECTD4	chr12	112645734	C	C	T	p.R2546H
RNF10	chr12	120984294	C	C	T	p.R82C
RNF10	chr12	121000809	C	C	T	p.A397V
C12orf43	chr12	121442834	G	G	T	p.R142S

(*Continued*)

Table S1: *Continued*

Gene	Chr		Sequence			
		Position	Reference_Allele	Tumor_Seq_Allele1	Tumor_Seq_Allele2	HGVSp_Short
TMEM120B	chr12	122213624	C	C	A	p.P339Q
WDR66	chr12	122406015	G	G	A	p.C904Y
DHX37	chr12	125470724	G	G	A	p.S65L
ULK1	chr12	132399943	G	G	A	p.R529Q
DDX51	chr12	132628640	G	G	A	p.R68W
NPAP1	chr15	24922423	T	T	C	p.V470A
RYR3	chr15	33954463	T	T	C	p.Y1578H
SQRDL	chr15	45981315	C	C	A	p.P399T
LYSMD2	chr15	52029791	A	A	G	p.L10P
DAPK2	chr15	64204120	G	G	C	p.P340R
TRIP4	chr15	64710825	A	A	G	p.Q419R
FEM1B	chr15	68582682	T	T	A	p.L329H
CORO2B	chr15	69011138	G	G	A	p.V357M
CHRNB4	chr15	78927812	A	A	G	p.L58P
ABHD2	chr15	89698698	C	C	A	p.C157*
POLG	chr15	89867440	G	G	T	p.Y656*
KIF7	chr15	90174764	C	C	T	p.D1025N
OR4F15	chr15	102359189	T	T	C	p.L267P
OR2C1	chr16	3406863	G	G	A	p.G308E
CIITA	chr16	10995970	C	C	A	p.P186Q
ABCC6	chr16	16284190	C	C	T	p.R489Q
ATXN2L	chr16	28834831	C	C	A	p.P84Q
ZNF48	chr16	30409070	C	C	G	p.P167A
SRCAP	chr16	30719021	C	C	A	p.S207R
FBXL19	chr16	30939842	C	C	T	p.L248F
ADCY7	chr16	50334768	G	G	A	p.A407T
CNGB1	chr16	57950082	G	G	T	p.T723K
CDH5	chr16	66436634	C	C	A	p.H639Q
TMEM208	chr16	67262994	G	G	A	p.R167Q
SMPD3	chr16	68405943	G	G	A	p.R48W
DHODH	chr16	72055197	G	G	A	p.R231H
BCAR1	chr16	75269472	G	G	A	p.S488F
MLYCD	chr16	83933152	C	C	G	p.P135A
OSGIN1	chr16	83999180	C	C	A	p.Y334*
PIEZO1	chr16	88788690	G	G	A	p.R1631C
MYOM1	chr18	3067357	G	G	A	p.S1654L
MIB1	chr18	19399487	C	C	A	p.A570E
ARID3A	chr19	932638	C	C	T	p.R197W
ABCA7	chr19	1042089	G	G	A	p.R110H

(*Continued*)

Table S1: *Continued*

Gene	Chr		Sequence			
		Position	Reference_Allele	Tumor_Seq_Allele1	Tumor_Seq_Allele2	HGVSp_Short
DOT1L	chr19	2210725	G	G	A	p.G408S
MLLT1	chr19	6222272	AGG	AGG	—	p.S323del
AP1M2	chr19	10692221	C	C	T	p.G163D
TNPO2	chr19	12813700	G	G	A	p.L748F
RTBDN	chr19	12940667	C	C	T	p.G75R
ZNF728	chr19	23158534	T	T	G	p.Q535H
CEP89	chr19	33378681	C	C	T	p.G648R
ARHGAP33	chr19	36273696	G	G	A	p.R449H
ZNF565	chr19	36673496	G	G	A	p.R458C
FAM98C	chr19	38897682	G	G	A	p.A295T
EID2	chr19	40030137	C	C	T	p.E195K
LTBP4	chr19	41128353	C	C	T	p.P1155S
ITPKC	chr19	41223782	G	G	A	p.G248S
HNRNPUL1	chr19	41800534	C	C	G	p.C487W
ZNF526	chr19	42729726	C	C	T	p.R391C
SRRM5	chr19	44118011	G	G	C	p.E580Q
CEACAM19	chr19	45176119	C	C	T	p.P103S
KCNA7	chr19	49573408	G	G	C	p.A428G
BCL2L12	chr19	50173627	G	G	A	p.R279H
TSKS	chr19	50243382	A	A	T	p.L519H
KLK9	chr19	51509822	G	G	T	p.Q120K
SIGLEC7	chr19	51650561	G	G	A	p.G403D
CD33	chr19	51729058	G	G	T	p.X140_splice
ZNF579	chr19	56089324	G	G	T	p.A561D
ZNF544	chr19	58757698	C	C	A	p.A22E
ZNF584	chr19	58921324	C	C	A	p.S12*
NOP56	chr20	2633944	A	A	G	p.N38S
TMEM239	chr20	2797494	G	G	A	p.G189S
RRBP1	chr20	17600306	C	C	T	p.C783Y
ZNF337	chr20	25657149	A	A	G	p.S259P
TM9SF4	chr20	30745619	C	C	T	p.A451V
MYH7B	chr20	33585298	G	G	A	p.R1243H
ARFGEF2	chr20	47567872	T	T	C	p.Y97H
APCDD1L	chr20	57042388	G	G	A	p.A172V
TAF4	chr20	60639570	C	C	G	p.A433P
HSPA13	chr21	15753565	C	C	T	p.A109T
RRP1	chr21	45213238	C	C	T	p.R105C
LSS	chr21	47614555	G	G	A	p.S613F

Table S2: The specific somatic mutations found in pulmonary lesion

Gene	Chr		Sequence			
		Position	Reference_Allele	Tumor_Seq_Allele1	Tumor_Seq_Allele2	Coding change
PLCH2	chr1	2433678	C	C	T	p.A903V
NPHP4	chr1	5947508	G	G	A	p.R775W
SLC45A1	chr1	8390646	G	G	A	p.A365T
MTOR	chr1	11308004	A	A	T	p.L330M
VPS13D	chr1	12408889	C	C	A	p.L3027I
HTR6	chr1	20005112	C	C	T	p.A256V
ZBTB40	chr1	22828932	A	A	G	p.R389G
MATN1	chr1	31188900	C	C	T	p.G355R
SYNC	chr1	33160901	G	G	T	p.D266E
ZSCAN20	chr1	33957137	C	C	A	p.L427I
KIF2C	chr1	45225971	G	G	A	p.A463T
POMGNT1	chr1	46663450	G	G	T	p.A15D
LEPR	chr1	66074550	G	G	A	p.R573H
C1orf173	chr1	75086457	C	C	T	p.E321K
SORT1	chr1	109884677	G	G	T	p.A356E
KCNC4	chr1	110754623	G	G	T	p.G168C
HRNR	chr1	152191964	C	C	G	p.S714T
FLG2	chr1	152327372	G	G	A	p.H964Y
FMOD	chr1	203317277	G	G	A	p.P41L
LAMB3	chr1	209811303	G	G	T	p.L108M
DNAH14	chr1	225533843	C	C	A	p.L3527I
ZNF678	chr1	227842419	C	C	A	p.D156E
ZNF678	chr1	227843089	A	A	G	p.K380E
FAM89A	chr1	231175746	C	C	T	p.G71D
OR6F1	chr1	247875319	C	C	T	p.V247M
OR11L1	chr1	248005196	C	C	G	p.M1?
KLHL29	chr2	23914746	G	G	A	p.E428K
MFSD2B	chr2	24233004	C	C	A	p.P13Q
DPYSL5	chr2	27150170	T	T	C	p.F157S
EIF2B4	chr2	27590987	G	G	A	p.H224Y
FOXN2	chr2	48573786	C	C	A	p.H145N
DYSF	chr2	71795443	G	G	A	p.D947N
CCT7	chr2	73478489	C	C	T	p.R447C
RANBP2	chr2	109382295	T	T	C	p.I1767T
MGAT5	chr2	135095831	C	C	T	p.A216V
ORMDL1	chr2	190640307	T	T	C	p.I104V
PTPRN	chr2	220161748	T	T	C	p.E732G
KIF1A	chr2	241661994	G	G	C	p.P1515R
BHLHE40	chr3	5021993	A	A	G	p.Y53C
CMTM8	chr3	32280541	G	G	C	p.S26T

(*Continued*)

Table S2: *Continued*

Gene	Chr		Sequence			
		Position	Reference_Allele	Tumor_Seq_Allele1	Tumor_Seq_Allele2	Coding change
ALS2CL	chr3	46717111	G	G	T	p.T751K
AMT	chr3	49455341	T	T	A	p.R315W
PPM1M	chr3	52282670	A	A	T	p.E99V
RFT1	chr3	53154034	A	A	G	p.F188L
CLDND1	chr3	98237751	G	G	T	p.S150R
TMPRSS7	chr3	111795757	G	G	A	p.G538R
RPN1	chr3	128341243	C	C	T	p.A469T
EFCC1	chr3	128721076	G	G	T	p.R202L
MRAS	chr3	138121031	A	A	G	p.S183G
RASA2	chr3	141291489	C	C	T	p.T403I
RASA2	chr3	141327518	T	T	C	p.L735P
B3GALNT1	chr3	160803554	T	T	C	p.H330R
GP5	chr3	194117949	G	G	A	p.P355S
SH3TC1	chr4	8229058	C	C	A	p.A546D
ZNF518B	chr4	10446539	G	G	T	p.H472N
KIAA1211	chr4	57179442	T	T	C	p.I145T
POLR2B	chr4	57873077	G	G	A	p.R438Q
CFI	chr4	110687775	TGTTGACAG	TGTTGACAG	—	p.Y85_Q88delins*
RNF150	chr4	142053793	G	G	T	p.A57D
FREM3	chr4	144618459	C	C	T	p.A1124T
TTC29	chr4	147858746	G	G	A	p.A59V
C4orf45	chr4	159836442	A	A	C	p.S142R
FAM50B	chr6	3850542	G	G	C	p.R166P
GFOD1	chr6	13365340	T	T	C	p.N270D
RAB44	chr6	36693650	C	C	T	p.R765W
TAF8	chr6	42025130	C	C	T	p.P123L
DLK2	chr6	43418566	C	C	T	p.R288Q
TMEM63B	chr6	44116297	G	G	A	p.R390H
TCTE1	chr6	44249900	C	C	T	p.A415T
TBX18	chr6	85447012	G	G	T	p.N405K
MDN1	chr6	90424459	G	G	A	p.S2291L
NKAIN2	chr6	124979462	C	C	A	p.T135K
HEY2	chr6	126080859	G	G	A	p.V309I
EPB41L2	chr6	131191262	G	G	A	p.S683L
TIAM2	chr6	155503389	C	C	A	p.R913S
TTYH3	chr7	2671886	C	C	T	p.R33W
VWDE	chr7	12428903	G	G	A	p.Q109*
KIAA0895	chr7	36374693	C	C	T	p.R321H
ZNF680	chr7	63982167	A	A	T	p.F322Y

(*Continued*)

Table S2: *Continued*

Gene	Chr		Sequence			
		Position	Reference_Allele	Tumor_Seq_Allele1	Tumor_Seq_Allele2	Coding change
ZNF92	chr7	64864312	A	A	G	p.K429E
CLIP2	chr7	73790928	C	C	A	p.H733N
ABCB1	chr7	87190672	A	A	G	p.L245S
AKAP9	chr7	91630964	C	C	A	p.A578E
SAMD9	chr7	92732094	G	G	A	p.A1106V
ZAN	chr7	100349694	G	G	A	p.V656I
MUC17	chr7	100682528	A	A	G	p.T2611A
KBTBD11	chr8	1950745	G	G	A	p.G463R
NKX3-1	chr8	23540266	G	G	T	p.T46K
NEFM	chr8	24771764	A	A	G	p.E153G
CHD7	chr8	61750681	G	G	A	p.G1467E
PAG1	chr8	81905455	G	G	A	p.P3L
KLF10	chr8	103664081	G	G	T	p.A160D
BAI1	chr8	143558854	A	A	G	p.N444S
EPPK1	chr8	144943476	G	G	A	p.Q1316*
VPS28	chr8	145649371	C	C	T	p.A201T
AQP3	chr9	33442469	G	G	C	p.D180E
KIAA1045	chr9	34977582	CAG	CAG	—	p.S354del
CA9	chr9	35677829	C	C	T	p.R295C
IGFBPL1	chr9	38413350	C	C	T	p.V191I
TRPM3	chr9	73213400	G	G	A	p.R983C
SEMA4D	chr9	92002472	G	G	T	p.P387T
NUTM2F	chr9	97081247	G	G	A	p.R591W
IKBKAP	chr9	111688835	T	T	C	p.E145G
ASTN2	chr9	119568025	T	T	A	p.K710M
SNAPC4	chr9	139277663	G	G	T	p.A653E
EGFL7	chr9	139563125	G	G	A	p.R66Q
ANKRD26	chr10	27322197	T	T	C	p.E1255G
MAP3K8	chr10	30739268	G	G	A	p.A196T
GDF10	chr10	48428943	C	C	T	p.A315T
SFTPD	chr10	81702245	G	G	A	p.P111L
ANKRD2	chr10	99340637	C	C	A	p.A188D
SCD	chr10	102108097	C	C	T	p.L102F
SEC31B	chr10	102248652	G	G	T	p.A1084E
VWA2	chr10	116046150	C	C	T	p.R484W
HRAS	chr11	534286	C	C	G	p.G13R
MUC2	chr11	1088774	T	T	A	p.C1187S
AMPD3	chr11	10517193	A	A	G	p.E457G
MRGPRX2	chr11	19077783	C	C	T	p.G56D

(*Continued*)

Table S2: *Continued*

Gene	Chr		Sequence			
		Position	Reference_Allele	Tumor_Seq_Allele1	Tumor_Seq_Allele2	Coding change
ALX4	chr11	44297028	C	C	T	p.R216Q
MAPK8IP1	chr11	45921740	T	T	A	p.L87M
CLP1	chr11	57427456	C	C	T	p.R170W
CTNND1	chr11	57577652	G	G	A	p.R836Q
HNRNPUL2	chr11	62484569	A	A	G	p.Y625H
ATG2A	chr11	64675268	A	A	G	p.V820A
KAT5	chr11	65482056	C	C	T	p.R261W
CCDC85B	chr11	65658658	T	T	C	p.L135P
TCIRG1	chr11	67808784	T	T	C	p.F16L
MSANTD4	chr11	105880515	T	T	C	p.E262G
HTR3B	chr11	113802517	T	T	A	p.M99K
GLB1L3	chr11	134180501	C	C	A	p.T379K
COL2A1	chr12	48387599	C	C	T	p.G306D
KMT2D	chr12	49416495	T	T	C	p.I5406V
SCN8A	chr12	52056780	A	A	G	p.E60G
SMARCC2	chr12	56563410	T	T	C	p.E842G
WIF1	chr12	65514884	G	G	A	p.P30S
CPM	chr12	69326541	T	T	C	p.E26G
ZFC3H1	chr12	72017964	G	G	A	p.Q1476*
LRRIQ1	chr12	85449325	G	G	T	p.E252*
FGD6	chr12	95603423	G	G	A	p.A546V
IKBIP	chr12	99019875	T	T	C	p.K323E
STAB2	chr12	104054149	T	T	A	p.V592D
FAM216A	chr12	110924514	C	C	A	p.N204K
SETD1B	chr12	122242676	C	C	G	p.H11Q
HIP1R	chr12	123338599	A	A	G	p.Q196R
SBNO1	chr12	123812517	G	G	T	p.P452T
DNAH10	chr12	124258809	C	C	T	p.Q146*
SFSWAP	chr12	132281845	C	C	T	p.S938L
ARHGEF40	chr14	21555241	T	T	C	p.L1407P
SALL2	chr14	21992261	G	G	T	p.S534*
SLC22A17	chr14	23817766	C	C	T	p.R214Q
ZFYVE1	chr14	73444637	C	C	T	p.G545R
FAM181A	chr14	94394681	C	C	T	p.A79V
DICER1	chr14	95579535	G	G	A	p.P645L
DYNC1H1	chr14	102481607	G	G	A	p.A2394T
AHNAK2	chr14	105411399	T	T	G	p.E3463D
AHNAK2	chr14	105419924	C	C	T	p.D622N
EIF2AK4	chr15	40268674	—	—	CG	p.S627Rfs*12

(*Continued*)

Table S2: *Continued*

Gene	Chr		Sequence			
		Position	Reference_Allele	Tumor_Seq_Allele1	Tumor_Seq_Allele2	Coding change
SPTBN5	chr15	42158408	G	G	T	p.A2217E
RFX7	chr15	56388427	C	C	T	p.G500E
ZNF280D	chr15	56946415	T	T	C	p.T732A
FOXB1	chr15	60297526	C	C	T	p.P122S
STRA6	chr15	74481603	G	G	C	p.L354V
MPI	chr15	75183897	T	T	C	p.S108P
ZZEF1	chr17	4005620	T	T	C	p.T555A
SMTNL2	chr17	4498516	G	G	A	p.E295K
MYH13	chr17	10227395	C	C	T	p.D960N
DNAH9	chr17	11607560	T	T	A	p.V1731E
SREBF1	chr17	17720013	G	G	A	p.A632V
ULK2	chr17	19699009	T	T	C	p.Q676R
TAOK1	chr17	27869770	C	C	A	p.H912Q
PSMD11	chr17	30771555	C	C	T	p.A5V
GRN	chr17	42426863	G	G	A	p.G70S
SNF8	chr17	47010578	G	G	T	p.Q185K
TMEM104	chr17	72815982	G	G	A	p.A244T
GGA3	chr17	73235939	C	C	T	p.G505D
LLGL2	chr17	73564583	G	G	C	p.V355L
PRSS57	chr19	687122	G	G	A	p.P150S
SBNO2	chr19	1114389	G	G	C	p.R640G
EFNA2	chr19	1298588	C	C	T	p.R165*
UBXN6	chr19	4452441	C	C	T	p.V121M
ZNF560	chr19	9577276	G	G	A	p.R783C
C19orf52	chr19	11039914	G	G	A	p.E107K
ZNF440	chr19	11941218	T	T	A	p.S42T
ZNF440	chr19	11941219	C	C	A	p.S42Y
C19orf67	chr19	14196112	G	G	T	p.L110I
OR7A10	chr19	14952117	G	G	T	p.D191E
OR7A17	chr19	14991800	A	A	G	p.F123S
RASAL3	chr19	15565622	G	G	A	p.R602*
PLVAP	chr19	17476358	G	G	T	p.R306S
SLC27A1	chr19	17611333	A	A	T	p.N455Y
CTC-260F20.3	chr19	19646459	T	T	C	p.I221T
ZNF737	chr19	20728254	C	C	G	p.S252T
ZNF737	chr19	20728407	A	A	T	p.F201Y
ZNF626	chr19	20807769	A	A	C	p.I305R
ZNF714	chr19	21300156	G	G	A	p.R229K
ZNF729	chr19	22497166	C	C	G	p.A316G

(*Continued*)

Table S2: *Continued*

Gene	Chr		Sequence			
		Position	Reference_Allele	Tumor_Seq_Allele1	Tumor_Seq_Allele2	Coding change
ZNF99	chr19	22940705	A	A	G	p.V669A
ZNF728	chr19	23158703	A	A	G	p.L479P
ZNF91	chr19	23543056	T	T	G	p.T909P
ZNF91	chr19	23543065	T	T	C	p.R906G
ZNF675	chr19	23836540	T	T	C	p.K399E
ZNF681	chr19	23926665	G	G	T	p.Q563K
ZNF30	chr19	35435101	A	A	T	p.T412S
ZNF829	chr19	37406711	A	A	C	p.I49S
LTBP4	chr19	41128518	G	G	T	p.G1210C
ZNF155	chr19	44501504	C	C	A	p.R510S
FBXO46	chr19	46215396	G	G	A	p.S453L
ZC3H4	chr19	47575639	G	G	A	p.T591M
DKKL1	chr19	49868848	A	A	G	p.E89G
CD33	chr19	51729058	G	G	T	p.X140_splice
ZNF845	chr19	53855552	T	T	C	p.C542R
LENG9	chr19	54973704	A	A	G	p.S358P
U2AF2	chr19	56180541	C	C	A	p.S346R
ZNF444	chr19	56658500	G	G	A	p.A74T
ZNF71	chr19	57132830	T	T	A	p.L59M
ZNF544	chr19	58773224	A	A	T	p.T418S
ZNF544	chr19	58773232	A	A	T	p.R420S
ADORA2A	chr22	24836610	T	T	C	p.L131P
TMPRSS6	chr22	37485763	G	G	T	p.H240N
SHANK3	chr22	51137156	G	G	A	p.V529M
TLR8	chrX	12938469	C	C	A	p.T437N
PHKA2	chrX	18942641	G	G	T	p.F524L
NYX	chrX	41333714	C	C	A	p.N336K
PRICKLE3	chrX	49040227	T	C	C	p.E91G
PPP1R3F	chrX	49126721	G	G	A	p.R130H
MSN	chrX	64951013	G	G	A	p.R171Q
AWAT1	chrX	69456942	G	G	A	p.V102I
BRWD3	chrX	79938082	T	T	A	p.I1427F
PABPC5	chrX	90691022	A	A	G	p.Y149C
ESX1	chrX	103499043	C	C	T	p.E100K
FMR1	chrX	147024739	C	C	A	p.T455K
PLXNB3	chrX	153035386	C	C	A	p.Q564K
PAPD7	chr5	6737706	G	G	A	p.R17Q
NIPBL	chr5	37020559	A	A	—	p.X1671_splice
ADAMTS6	chr5	64492921	T	T	C	p.N878S

(*Continued*)

Table S2: *Continued*

Gene	Chr	Position	Sequence			Coding change
			Reference_Allele	Tumor_Seq_Allele1	Tumor_Seq_Allele2	
ERBB2IP	chr5	65364707	C	C	A	p.S1212R
DMXL1	chr5	118469580	C	C	A	p.T654K
APBB3	chr5	139938282	A	A	G	p.L457P
PCDHGC5	chr5	140870581	G	G	T	p.V592L
FAT2	chr5	150901442	T	T	C	p.E3571G
SLIT3	chr5	168175400	G	G	A	p.P726L
RANBP17	chr5	170319547	A	A	G	p.K138R
SLC34A1	chr5	176823987	T	T	C	p.L443P
ZC3H13	chr13	46563120	T	T	C	p.R353G
LRCH1	chr13	47127622	C	C	A	p.H31N
THSD1	chr13	52972055	G	G	T	p.S111R
COL4A1	chr13	110859028	C	C	G	p.G281A
ARHGEF7	chr13	111806328	T	T	A	p.L81Q
RAB11FIP3	chr16	555594	G	G	A	p.R489H
TBL3	chr16	2025427	G	G	A	p.A265T
PKMYT1	chr16	3022991	A	A	G	p.L488P
GPR114	chr16	57597763	G	G	A	p.G101R
ACD	chr16	67693896	G	G	T	p.T137K
C16orf86	chr16	67701410	C	C	A	p.P105Q
CDH1	chr16	68853188	G	G	A	p.R524Q
ZFHX3	chr16	72828084	C	C	T	p.D2833N
RFWD3	chr16	74662416	G	G	T	p.P635T
TLDC1	chr16	84514232	C	C	T	p.C387Y
RBBP8	chr18	20596880	G	G	A	p.C816Y
FASTKD5	chr20	3127843	A	A	G	p.L625P
RRBP1	chr20	17640684	C	C	T	p.V157M
C20orf26	chr20	20269379	A	A	T	p.R975*
RALGAPA2	chr20	20569939	A	A	T	p.H804Q
TTI1	chr20	36611971	C	C	T	p.V1053M
OSER1	chr20	42825712	C	C	T	p.A287T
LAMA5	chr20	60898590	G	G	A	p.R1996C
BIRC7	chr20	61867740	C	C	A	p.P98T
SON	chr21	34923272	A	A	T	p.T579S
PRDM15	chr21	43281809	A	A	G	p.F252L

Table S3: The mutations shared in both lesions

Gene	Chr	Position	Sequence			Coding change
			Reference_Allele	Tumor_Seq_Allele1	Tumor_Seq_Allele2	
ZSCAN20	chr1	33957137	C	C	A	p.L427I
OR6F1	chr1	247875319	C	C	T	p.V247M
ORMDL1	chr2	190640307	T	T	C	p.I104V
CFI	chr4	110687775	TGTTGACAG	TGTTGACAG	—	p.Y85_Q88delins*
HRAS	chr11	534286	C	C	G	p.G13R
HTR3B	chr11	113802517	T	T	A	p.M99K
CD33	chr19	51729058	G	G	T	p.X140_splice
PRICKLE3	chrX	49040227	T	C	C	p.E91G
